# Hyper-parameter tuned deep learning approach for effective human monkeypox disease detection

**DOI:** 10.1038/s41598-023-43236-1

**Published:** 2023-09-23

**Authors:** Neeraj Dahiya, Yogesh Kumar Sharma, Uma Rani, Shekjavid Hussain, Khan Vajid Nabilal, Anand Mohan, Nasratullah Nuristani

**Affiliations:** 1 Department of Computer Science and Engineering, SRM University Delhi-NCR, Sonipat, Haryana India; 2 Department of Computer Science and Engineering, Koneru Lakshmaiah Education Foundation, Vaddeswaram, Guntur, Andhra Pradesh India; 3 Department of Computer Science and Engineering, World College of Technology and Management, Gurugram, Haryana 122413 India; 4 Department of Computer Science and Engineering, Shri Jagdishprasad Jhabarmal Tibrewala University, Jhunjhunu, Rajasthan India; 5 Department of Computer Science and Engineering, Dhole Patil College of Engineering, Wagholi, Pune, Maharashtra 412207 India; 6 Department of Physics, Kunwar Singh College, Darbhanga, Bihar India; 7 Department of Spectrum Management, Afghanistan Telecommunication Regulatory Authority, Kabul, 2496300 Afghanistan

**Keywords:** Diseases, Engineering

## Abstract

Human monkeypox is a very unusual virus that can devastate society. Early identification and diagnosis are essential to treat and manage an illness effectively. Human monkeypox disease detection using deep learning models has attracted increasing attention recently. The virus that causes monkeypox may be passed to people, making it a zoonotic illness. The latest monkeypox epidemic has hit more than 40 nations. Computer-assisted approaches using Deep Learning techniques for automatically identifying skin lesions have shown to be a viable alternative in light of the fast proliferation and ever-growing problems of supplying PCR (Polymerase Chain Reaction) Testing in places with limited availability. In this research, we introduce a deep learning model for detecting human monkeypoxes that is accurate and resilient by tuning its hyper-parameters. We employed a mixture of convolutional neural networks and transfer learning strategies to extract characteristics from medical photos and properly identify them. We also used hyperparameter optimization strategies to fine-tune the Model and get the best possible results. This paper proposes a Yolov5 model-based method for differentiating between chickenpox and Monkeypox lesions on skin pictures. The Roboflow skin lesion picture dataset was subjected to three different hyperparameter tuning strategies: the SDG optimizer, the Bayesian optimizer, and Learning without Forgetting. The proposed Model had the highest classification accuracy (98.18%) when applied to photos of monkeypox skin lesions. Our findings show that the suggested Model surpasses the current best-in-class models and may be used in clinical settings for actual Human Monkeypox disease detection and diagnosis.

## Introduction

The orthopoxvirus that causes monkeypox is a member of the Poxviradea family and is notorious for having very complex double-stranded DNA. This virus is becoming increasingly important as a cause of human illness. Smallpox eradication zones are not immune to human monkeypox infections. The monkeypox virus may quickly jump from mammal to animal. The monkeypox virus has been isolated twice. However, its natural host is still a mystery. Transmission from human to human has shown a 12-day incubation time for the monkeypox virus^1^. Direct contact with the exudate or crust material of the lesion is thought to be the primary transmission mode. At the same time, the virus might also be spread through respiratory secretions and saliva. The virus might also spread through bodily secretions. The monkeypox virus has many of the morphological characteristics of other orthopoxviruses. It is around 200–250 nm in size and has an envelope, surface tubules, and a dumbbell-shaped core. The core section of the monkeypox virus genome, which encodes structural proteins and critical enzymes, shares 96.3% sequence similarity with the variola virus; however, the region of the genome encodes virulence factors and host range factors is very different. Monkeypox has a case fatality rate between that of variola minor (1%) and variola major (30%)^2^.

In recent years, there has been a dramatic increase in confirmed instances of human monkeypox virus and its geographic expansion as protection to smallpox immunization declines. With a mortality rate of 6%, the 2017 monkeypox epidemic in Nigeria was the worst in the West African clade. The United Kingdom (UK) saw two instances of monkeypox imported by Nigerian persons in September 2018, with one case being the source of nosocomial infections impacting healthcare personnel.

Furthermore, in 2003, mice smuggled from Ghana and kept with prairie dogs brought the monkeypox virus to the United States of America (USA), where it was eventually transmitted to people. The only two known are West African and Central African clades of monkeypox viruses. There have been no reports of human-to-human transmission in the former. Still, communication within the Central African clade has been recorded in the latter, and the case fatality rate is 11%. The virus that caused the outbreak in the United States is known as the West African variety.

In contrast, it has been linked to higher rates of death and transmission between humans and higher viral loads in the blood compared to the West African clade. No casualties were reported, although adults were hit harder than children were in the United States. Death rates in Africa range from 1.5% to 17%, with children bearing the brunt of this West African clade observed in Nigeria^3^.

This factor may reflect the decline in community immunity since smallpox immunization was discontinued. Monkeypox can also be passed from mother to child through the placenta (resulting in congenital monkeypox) or by prolonged, intimate contact after delivery. Further research is required to comprehend this danger fully.

Anything from a handful to thousands of lesions has been found. In extreme circumstances, the lesions may join together and cause significant areas of skin to flake off. Typically, the duration of symptoms associated with monkeypox is between two and four weeks, and the disease resolves on its own. Children are disproportionately represented in cases of severe illness, which are linked to factors such as the duration and severity of viral exposure, the general health of the patient, and the presence and kind of sequelae. Impairments to the immune system underneath the surface may have disastrous consequences. Although the smallpox vaccine proved effective in the past, vaccination programs have ended worldwide following the disease's eradication^4^.

### Diagnosis

Conditions should all be evaluated in the clinical differential diagnosis of a rash. One clinical sign that may help differentiate monkeypox from chickenpox or smallpox is the presence of lymphadenopathy in the prodromal phase of the illness. If health professionals suspect monkeypox, they must obtain a representative sample and send it to a lab with the necessary equipment. The laboratory test and specimen used to confirm monkeypox determine its severity and whether or not it may be treated. Important patient information that should be included with specimens includes (a) the patient’s age, (b) the date of the beginning of fever, (c) the date of specimen collection, (d) the patient’s present state (stage of rash), and (e) the date of collection^5^. The critical contribution of this research is as follows:

- This study aims to provide a method for automating the detection and categorization of monkeypoxes. Initial training pictures for the proposed Model were created using autoargumention, which can identify the textural link between image pixels.
- In this study, the Roboflow Monkeypox illness dataset was used.
- The modified Yolov5 Model can differentiate monkeypox from healthy tissue.
- Existing approaches were implemented in Google Cololab alongside the proposed method for comparison; they included the Yolov5 model and SDG optimizer, as well as a hybrid version of Learning without Forgetting.
- The suggested Model outperforms the state-of-the-art in precision, Accuracy, and sensitivity.

The full study should be written as follows: “Related work” section discusses previous research, “Dataset” section describes the Dataset, “Methods” section describes the proposed method, “Results and discussion” section describes the experimental results and analysis, and “Discussion” section discusses the conclusion and future works.

## Related work

Matuszewski et al.^6^ studied virus identification, segmentation, classification, and novelty detection; a dataset of transmission electron microscopy (TEM) images has been employed in this article. It was also shown that tiny hand-crafted networks perform well when trained from scratch. However, transfer learning is critical for the decent performance of extensive networks when dealing with restricted datasets.

Dwivedi et al.^7^ propose using deep learning models to automate the diagnosis process for Monkey Pox, a new pandemic threat. It compares performance between ResNet50, EfficientNetB3 and EfficientNetB7 algorithms on a limited Dataset with promising results. The paper’s results suggest that ResNet50 achieved an accuracy of 83%, EfficientNetB3 produced 87%, and EfficientNetB7 initially had a good effect, but as more epochs were added, its Accuracy decreased dramatically.

Singh et al.^8^ discuss using deep learning techniques for the automated identification of skin lesions associated with monkeypox. The results showed that GoogLeNet achieved 88.27% among all four tested models, suggesting its potential to aid early diagnosis and surveillance efforts against this disease. By using computer-assisted detection methods such as these, it may be possible to reduce misdiagnosis rates and improve public health outcomes related to monkeypox outbreaks worldwide. This study showed that deep learning techniques could accurately identify skin lesions associated with monkeypox. GoogLeNet achieved the highest Accuracy (88.27%) among all four models tested, suggesting its potential to aid early diagnosis and surveillance efforts against this disease. This research provides evidence for further exploration into computer-assisted detection methods to reduce misdiagnosis rates and improve public health outcomes related to monkeypox outbreaks worldwide.

Sahin et al.^9^ proposed a system based on MobileNetv2 and achieved 91.11% accuracy in classifying images. This will help reduce the spread rate by encouraging infected individuals to act rapidly and seek expert advice for definitive diagnosis. It also has potential applications in other skin diseases, as it can be trained using different datasets with a similar deep transfer learning approach. The results reported in Iit outperformed other methods concerning metrics when tested using the MSLD database (a publicly available dataset).

Rabaan et al.^10^ discuss the recent monkeypox outbreak so that computer-assisted detection could benefit surveillance. It also introduces a new dataset called Monkeypox Skin Lesion Dataset (MSLD), which consists of skin lesion images of monkeypox, chicken pox and measles collected from websites & news portals, etc., with deep learning models used to classify them accurately.

Benges et al.^11^) examine public attitudes on the latest monkeypox outbreak to aid decision-makers. It was collecting tweets in many languages on monkeypox, performing sentiment analysis with VADER and TextBlob, and creating, testing, and assessing 56 classification models for performance evaluation based on Accuracy, F1 Score, etc. Throughout the scope of this article, we have collected approximately 500,000 tweets from across many languages that discuss the Twitter post on monkeypox. For positive, negative, and neutral feelings, it used VADER and TextBlob annotation methods. For vocabulary normalization, the researchers created 56 classification models and included vectorizations.

Human Monkeypox Detection (HMD) is a new detection system discussed by Saleh et al.^12^ that uses AI methods for the early identification of monkeypox patients. This HMD comprises two primary phases: the Selection Phase, which aims to pick the best characteristics, and the Detection Phase, which uses a weighted vote system to merge three different diagnostic algorithms into a single diagnosis using an Ensemble Diagnosis model. Two essential parts, "Selection" and "Detection," make up the proposed Human Monkeypox Detection (HMD) technique. Before moving on to the DP phase of learning an Ensemble Diagnosis model, the Selection phase uses an Improved Binary Chimp Optimization algorithm to choose valuable features. The Filter Selection Layer in this IBCO algorithm is responsible for rapidly selecting significant components, and the Wrapper Selection Layer is in charge of constructing the initial population of the Binary Chimp Optimization Algorithm.

Haque et al.^13^ present a study on the current monkeypox outbreak. Panel 2 detected the 2022 MPXV B1 lineage and its descendant lineages. Both panels showed high specificity for identifying different monkeypox virus strains with no cross-reactivity from other viruses in real-time PCR tests. The paper only focused on the detection and differentiation of MPXV, not other poxviruses. It did not evaluate how well these assays work in clinical samples or their potential for use as a diagnostic tool.

Shahyeez Ahamed et al.^14^ conducted a comprehensive literature search and study of the evolution and mutation of host cells, regulatory policies, vaccination and treatment advancements, and historical context. Recent research has revealed that the currently circulating pox strain contains several genomic alterations associated with APOBEC3 regulation. Transmission of the now prevalent pox has also been hypothesized to occur via sex. The use of vaccinations and the rapid development of an anti-pox medication are both essential needs. More research is required to guarantee the elimination of epidemics.

In their review, Nieto-Chaupis et al.^15^ discuss the recent epidemic of monkeypox, its likely causes, and the virus's transmission, pathogenesis, and clinical presentation. Moreover, we explore whether or not the monkeypox virus might spread beyond Africa and become endemic there. Several measures have been taken to combat this epidemic, including active case identification, contact tracing, isolation, and postexposure vaccination, despite evidence suggesting that human-to-human transmission is still occurring and disconnected clusters persist.

In this study, Irmak et al.^16^ used four methods to detect Monkeypox virus (MPXV) DNA from 154 human samples. The first method was the Novaplex™ MPXV Assay, a real-time PCR assay that uses specific primers and probes to detect viral genetic material in clinical specimens. The second method was Bio-Speedy® Monkeypox Virus qPCR Kit, which utilizes real-time PCR technology with its own set of primers and probes explicitly designed for monkeypox detection.

Table 1 summarises the current work in the Monkeypox disease prediction as below.

**Table 1:** State-of-art Monkeypox disease detection approaches.

S. No	Author	Method	Dataset	Performance
1	Matuszewski et al.^6^	DenseNet201	1245 images of 22 different virus classes	93.1% Accuracy
2	Dwivedi et al.^7^	ResNet5 model	Kaggle Monkeypox dataset	87% Accuracy
3	Singh et al.^8^	GoogLeNet model	Kaggle dataset	88.27% Accuracy
4	Sahin et al.^9^	MobileNetv2 model	MSLD database	91.11% Accuracy
5	Rabaan et al.^10^	Pre-trained deep learning models	Monkeypox Skin Lesion Dataset (MSLD)	82.96(± 4.57%) Accuracy
6	Benges et al.^11^	SVM model	500,000 monkeypox Twitters	93.48% Accuracy
7	Saleh et al.^12^	Weighted Naïve Bayes (WNB)	Monkeypox dataset	92.56% Accuracy

The development of hyper-parameter tailored deep learning models for Human Monkeypox Disease Detection has come a long way. However, there are still certain knowledge gaps that need to be filled. Some of the biggest holes in the research are as follows.

- *Not enough commonly used datasets* Human Monkeypox Disease Detection lacks standard datasets. Generalizing the results to diverse populations and circumstances is challenging since most models have been built on short datasets with limited diversity.
- *Few available data from endemic areas* More research should be conducted in areas where the illness is endemic. This would allow us to create more precise, locally relevant models.
- *Poor generalizability* Many models have been produced in the lab but haven't been put to good use in the actual world. Therefore, models geared toward implementation in real clinical situations are required.
- *Insufficient interpretability* Given the complexity of deep learning models, they are sometimes called “black boxes” since it is impossible to determine the reasoning behind their diagnostic conclusions. To make these models more human-understandable, more study is required into explainable AI methods.
- *Unsatisfactory progress in disease pathology education* The pathogenesis of Human Monkeypox Disease is still poorly understood. The methods of disease transmission, genetic variables that increase vulnerability, and the immunological response to infection are all parts of this bigger picture. This information is necessary for the creation of efficient diagnosis and treatment methods.

These knowledge gaps must be filled to create effective, robust, and clinically applicable hyper-parameter-tailored deep learning models for Human Monkeypox disease detection.

## Material and methods

This section details the Dataset and method proposed in this research work.

### Dataset

**Figure 1:**
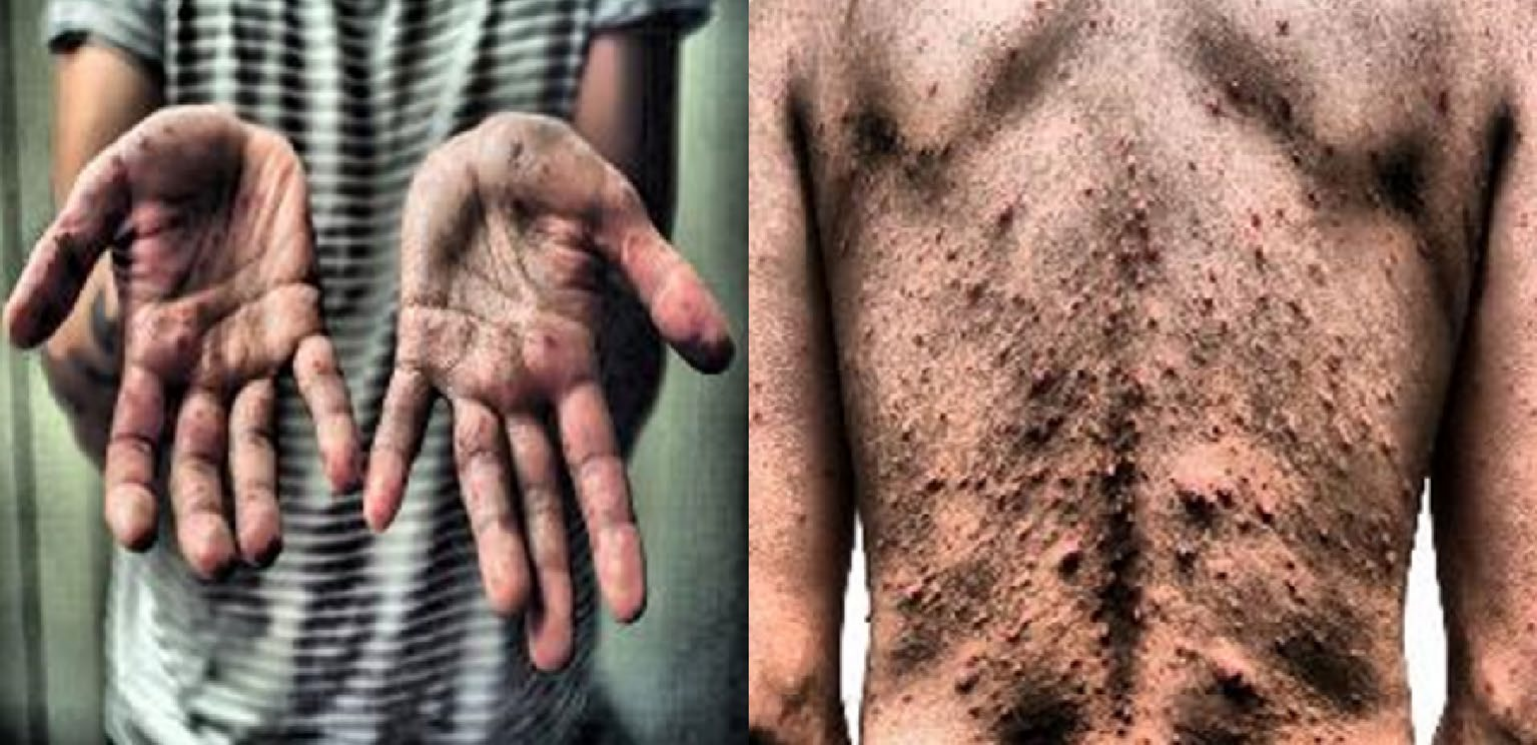
Train set images of Monkeypox Robolflow Dataset.

The Dataset has been the Roboflow Dataset Repository. The link to the prescribed Dataset is https://universe.roboflow.com/monkeypox-o7ktt/monkeypox-detection-lym6c. This Dataset consists of 971 images, with 849 pictures used as Training Set, 81 as Validation Set, and the remaining pictures used in Testing Set. There are some samples of Training images shown in Fig. 1 below:

#### Preprocessing

**Figure 2:**
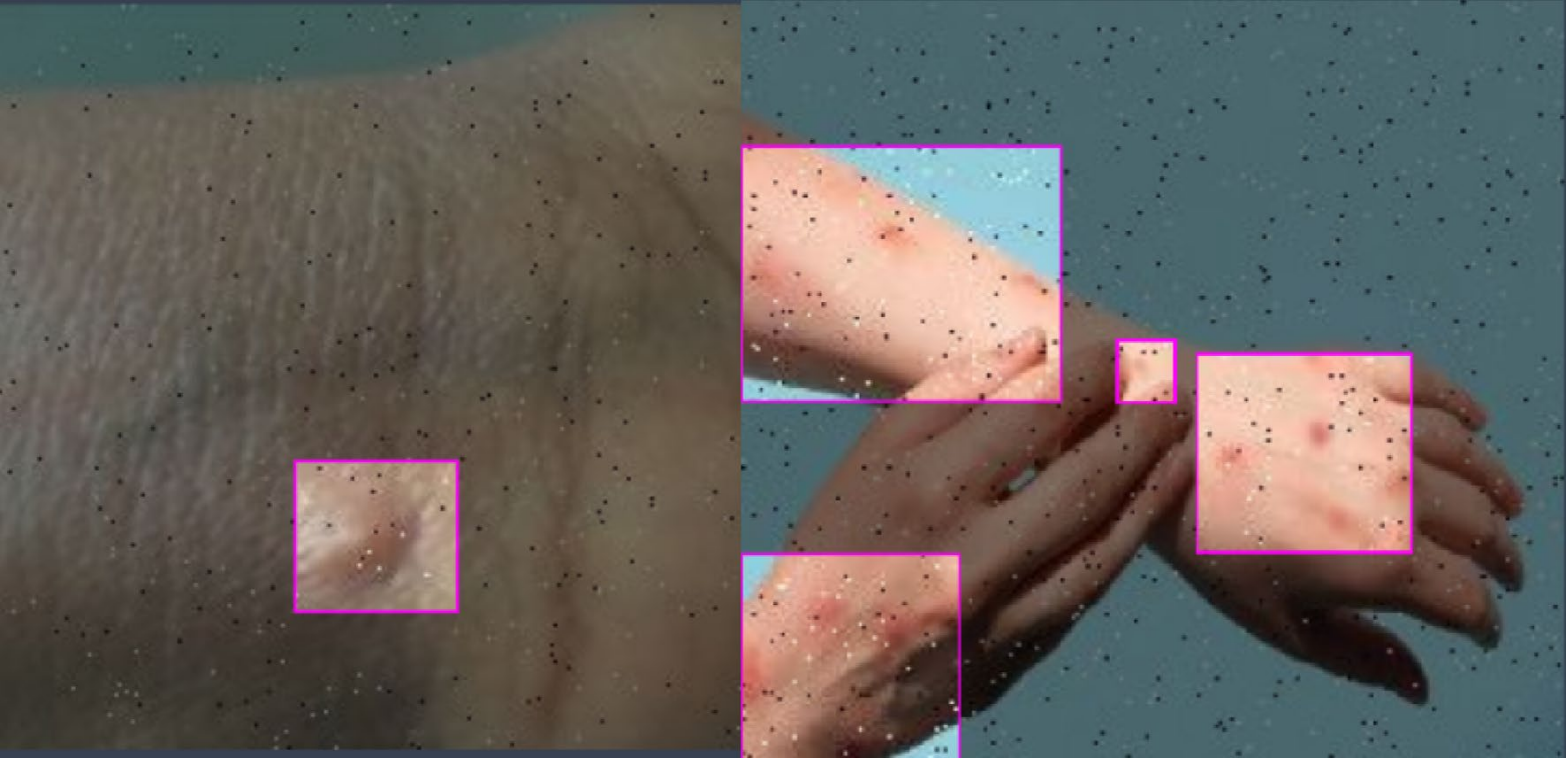
Image argumentation.

One example of a preprocessing phase is the usage of image transformations to ensure that the dataset is consistent across all three subsets. Two such examples are cropping photos statically or converting them to grayscale. The same preparation processes are used for all three testing phases (training, validation, and testing). Modifying the training photos ever-so-slightly can help to train with much more data. These occurrences are unique to the training set and should be ignored when testing and grading^17^. The ground truth photos from the validation and test sets are recommended when assessing. The authors of this article used the AutoAugment method, which automatically finds the optimal complement of augmentations for a given dataset. It shows that a tailored set of enhancements boosts the Model's performance. Figure 2 shows the image argumentation on the training set as below.

For computer vision tasks like classification, segmentation, and object identification, augmentations assist deep neural networks in resisting overfitting and enhancing performance. The best thing is that it is feasible to add picture augmentations to any computer vision pipeline without effort, thanks to libraries like Albumentations^18^. The parameter p determines the likelihood that each augmentation will alter the input picture. In addition, several enhancements allow to fine-tune how much an image is changed. A.RandomBrightnessContrast, for instance, accepts two parameters: brightness limit and contrast limit, which determine the relative size of the brightness and contrast changes, respectively. Larger values have a more noticeable effect on an image's appearance after enhancement^19^.

**Figure 3:**
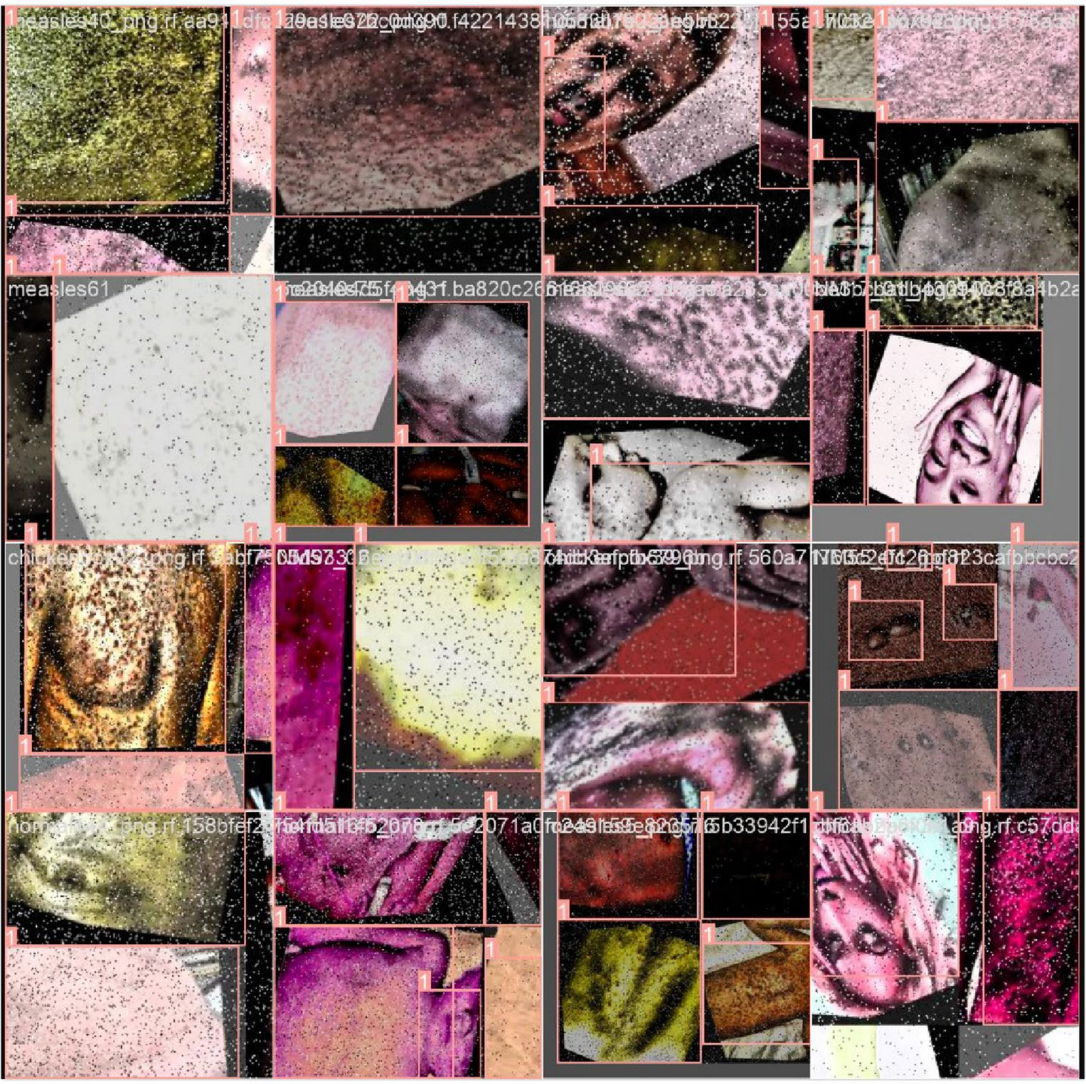
Training phase outcomes.

An enhancement's transformation magnitude is drawn from a normal distribution with the parameters brightness and contrast limits, as illustrated in Fig. 3. It implies that using the same input picture for many transform calls will provide different results each time.

### Methods

**Figure 4:**
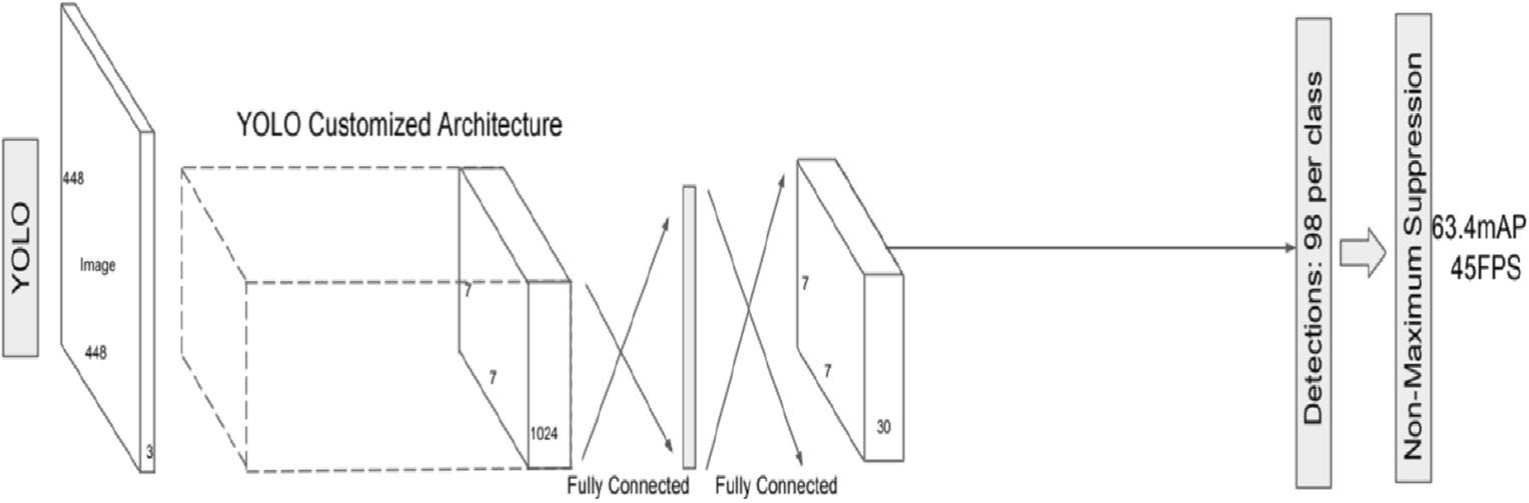
Yolov5 model.

The three main phases of conventional object detection methods are depicted in the following diagram. In the initial stage, potential regions are proposed. Perhaps containing items, these proposed regions are candidates. Typically, the number of such areas is in the thousands, say, 2000 or more^20^. Selective Search and EdgeBoxes are two examples of algorithms that may be used to propose regions. Several image descriptors, such as the histogram of oriented gradients (HOG), extract a feature vector of a specified length from each proposed part. The effectiveness of object detectors relies heavily on this feature vector. Even if an item is scaled or translated, the vector should still be able to represent it accurately. Deep neural networks have recently served as end-to-end learning instances in various contexts^21^. The picture of the patient may be used as input to a neural network, and the network can then provide an argument about whether or not the patient has a monkeypox. In a neural network, each node is a mathematical function that, given a set of numerical inputs via the edges, returns a set of numerical outputs via the output edges. During the training of a deep neural network, the parameters of the network are adjusted such that the mapping gets better over time. This process is a computationally demanding process that has recently benefited greatly from a variety of conceptual and engineering advances^22–26^. Figure 4 depicts the architecture of the Yolov5 Model as below.

This research aims to solve this issue by collecting and publicly sharing thousands of photos of healthy and ill humans. In this work, we provide the results of a convolutional neural network-based categorization of 971 photos of various human disorders. When given a set of 38 classes, our models are evaluated based on their Accuracy in predicting the disease. Our findings represent an early stage in developing a mobile phone-based diagnostic method for monkeypox. Object Detection with No Additional Steps Object detection models are one-stage if they do not employ an area proposal step and instead perform detection over a highly dense sample of locations. It is more common for inference in these models to be more rapid (possibly at the cost of performance). There is only one step in the YOLOv5 Model for detecting objects. The network draws on features from the entire image for each predicted bounding box. The image's bounding boxes are expected at once, regardless of category. In other words, the network makes decisions based on the entire images and the objects in the pictures.

### Proposed model

YOLO is a Convolutional Neural Network (CNN) that can identify objects in real time. The input photos are processed as organized data arrays, and CNNs are classifier-based systems that can identify patterns between them (view the image below). YOLO’s speed and Accuracy are both improvements over competing networks. During the testing, the Model may take in the entire photo, allowing for more accurate predictions based on the full context of the picture. Positive detections of whatever category the high-scoring area most closely matches are indicated. With a live traffic stream, for instance, YOLO may be utilized to identify certain car classes by analyzing which video areas perform best compared to known car types. In contrast to previous versions of YOLO, Yolov5 significantly enhances the Accuracy of real-time object recognition without raising inference costs^27–30^.

“Extended Efficient Layer Aggregation Network” (E-ELAN) refers to the computing block in the Yolov5 backbone. By employing “expand, shuffle, merge cardinality”, the E-ELAN architecture of Yolov5 enables the Model to learn better without losing the original gradient route through ongoing improvement of the network's learning capabilities. Model scaling, for instance, can enhance the Model's breadth, the number of stages and resolution. Conventional methods employing concatenation-based topologies (like ResNet or PlainNet) make it impossible to examine various scaling variables in isolation. Increases in model depth, for instance, alter a transition layer's input/output channel ratio, which may reduce the layer's hardware requirements.

For this reason, Yolov5 includes compound model scaling, which is a concatenation-based method. The ideal structure of the Model may be preserved together with the qualities it originally possessed using the compound scaling technique. This factor is also how the scaling of compound models works. For instance, modifying a computational block’s depth factor affects the block's output channel. Next, the width factor is scaled while maintaining the same degree of change on the intermediate levels^31^.

Despite its impressive results in VGG designs, using RepConv directly with ResNet or DenseNet results in a considerable drop in Accuracy. Planned re-parameterized convolution in Yolov5 uses RepConv without identity connection (RepConvN).

Here is a rundown of the YOLO (You Only Look Once) hyperparameters, along with some recommended tweaks:

- *Learning rate* Identifies the optimizer's step size and the rate at which the Model adapts to new data. Models can diverge if the learning rate is too high and converge slowly if it's too low. As the Model's performance on the validation set improves, the learning rate can be increased from the suggested starting value of 0.001.
- *Batch size* Set the number of photos to be processed during each training iteration. The variation of the gradient estimates can be decreased by increasing the batch size; however, this comes at the expense of increased memory usage and potential training delays. A batch size of 32 or 64 is a decent benchmark to work off of.
- *Epoch count* Iterations across the full Dataset performed by the training procedure. Underfitting occurs when there are too few epochs, whereas overfitting occurs when there are too many. It is suggested to begin with a small number of epochs (for example, 50) and keep an eye on the performance of the validation set.
- *Anchor boxes* YOLO employs anchor boxes to recognize objects of varied sizes and aspect ratios. The total number of anchor boxes and their average size significantly impact the Model's Accuracy. Try various anchor box sizes and aspect ratio combinations to identify the ideal configuration for unique Dataset.
- *Size of input image* Input picture size can affect the Model's efficiency and performance. However, increasing the amount of input requires more processing power and more time to train. It would help if it tried several input sizes to identify the optimal trade-off between precision and speed for specific application.
- *Dropout rate* To avoid overfitting, a regularization method called "dropout" involves randomly eliminating some neurons from the training set. The likelihood of a neuron being removed from the network at each iteration depends on the dropout rate. A dropout rate of 0.5 is suggested as a starting point, and it may be modified as the Model's performance on the validation set is evaluated.
- *Weight decay* Overfitting may be avoided using a regularization method that assigns penalties to high weight values. The weight decay hyperparameter controls the severity of this cost. It is advised to start with a weight decay of 0.0005 and fine-tune it depending on the Model's performance on the validation set.
- *Activation function* In the convolutional layers of YOLO, the LeakyReLU activation function is used. ReLU and sigmoid are two other activation functions that can be utilized instead. Try out a few distinct activation functions until it discover one that serves the purposes admirably.
- *Optimizer* Optimization techniques like stochastic gradient descent (SGD), Adam, and RMSprop are all options for YOLO. Try out a few distinct optimizers until it locate one that serves the needs perfectly.
- *Loss function* To fine-tune its Model, YOLO employs a mixed bag of localization and classification losses. Focal loss and IoU loss are two examples of alternative loss functions. It would help if it has been tried out several loss functions to see which one performs best for its application.

The combined Training approach retrains the whole network after inserting a new branch specifically for the new job. The ideal settings for the YOLO hyperparameters change from Dataset to Dataset and task to task. These numbers may not be the best for particular scenario and data collection, so keep that in mind. It is typically required to try out a range of parameters to observe how they influence the Model's efficiency. Finding the optimal hyperparameter values for a given job may be done using a grid or a random search. YOLO suggests an end-to-end neural network. The predictions in YOLO are made by a single completely connected layer, which is a significant improvement over existing algorithms.
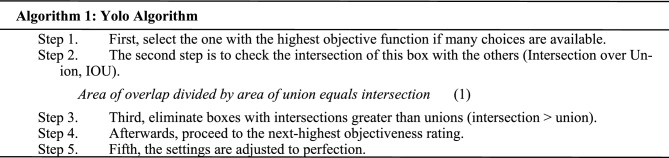


Everything that occurs in each given one of these N grids is the responsibility of that grid. So, these grids provide illness predictions for monkeypoxes based on information such as the label and presence probability of items within a particular cell and the bounding box coordinates of those objects relative to the cell's coordinates. Specifically, for Human Monkeypox Disease Detection, the steps would be.
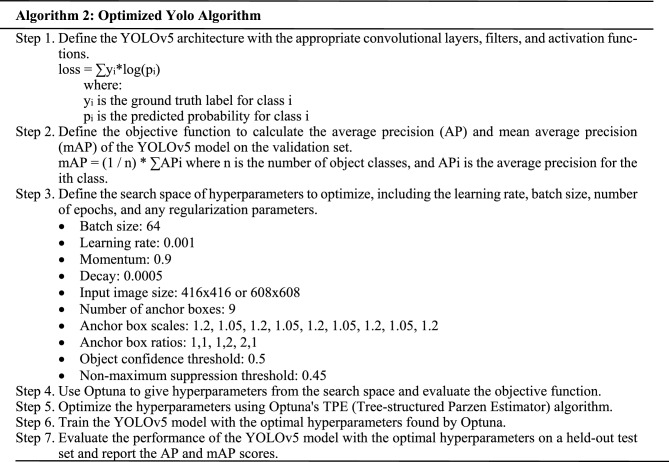


Optimization of the YOLOv5 model's hyperparameters based on Accuracy and speed of item detection on the provided Dataset would be the goal of Optuna's TPE method in YOLO Monkeypox Disease Detection. Training the YOLOv5 model on the Monkeypox Disease dataset would include the function taking in hyperparameters such as learning rate, batch size, number of epochs, anchor box size, etc. Mean average precision (mAP) is a statistic that measures a model's Accuracy by averaging its precision and recall.

## Results and discussion

### Experiment setting

The studies used a laptop with a 2.40 GHz Intel Core i3-4000 M CPU and 4 GB of RAM connected to the Collaboratory's Tesla K80 GPU. We employed the Keras and TensorFlow Python libraries as open-source DL software packages in our investigation. We also used the statistics package Scikit-learn to assess the performance metrics.

### Classification measure

The following parameters help better understand and analyze the Model and its performance.

- Accuracy:
  2 $$Accuracy = \frac{TP + TN}{{TP + TN + FP + FP}} = \frac{Correct\;predictions}{{Total\;predictions}}$$
- Precision:
  3 $$Precision = \frac{TP}{{TP + FP}} = \frac{Predictions\;actually\;positive}{{Total\;predicted\;positive}}$$
- Recall (TPR, sensitivity): It is calculated as:
  4 $$Recall = \frac{TP}{{TP + TN}} = \frac{Predictions\;actually\;positive}{{Total\;actual\;positive}}$$

### Experiment 1: Yolov5 model

In Experiment 1, the authors executed the basic Yolov5 on the prescribed Roboflow Monkeypox dataset on 100 different echos. Recall may be calculated for YOLO-based object identification by comparing the ground-truth bounding boxes of items in a picture with the predicted bounding boxes produced by the YOLO model. The prediction is deemed accurate if a projected bounding box overlaps a ground truth bounding box by more than some threshold (say, 0.5 for the popular IoU measure).

**Figure 5:**
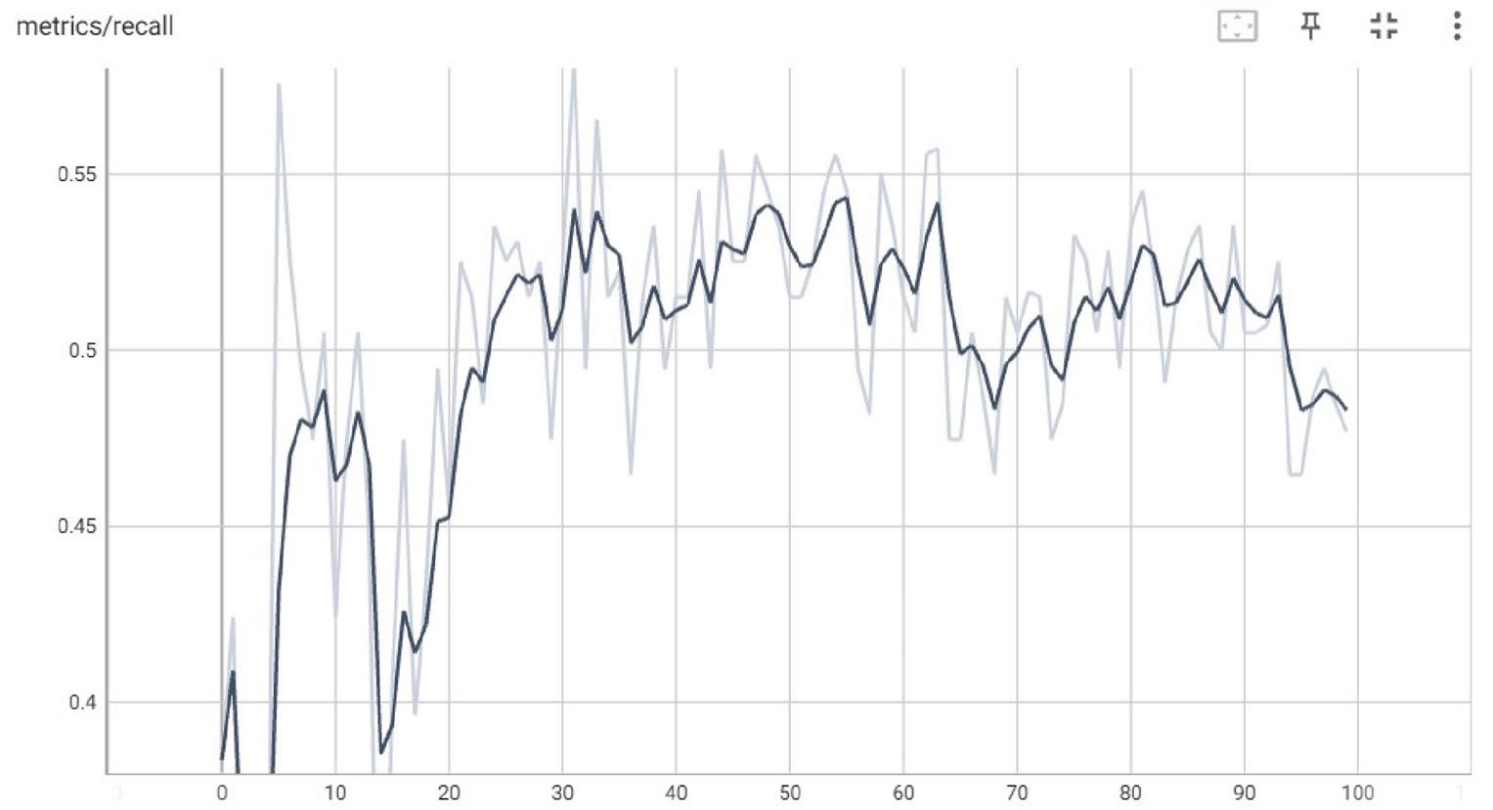
Recall gained by the basic Yolov5 model.

In many scenarios, it is preferable to have a high recall since missing an important instance might have dire repercussions. It is vital to establish a balance between these metrics based on the needs of the application since greater recall might come at the expense of reduced Accuracy (the proportion of projected positive occurrences that are positive). Figure 5 demonstrates the recall values at distant echos. The x-axis represents the echo value, and the y-axis represents the recall value gained by the basic Yolov5 Model.

**Figure 6:**
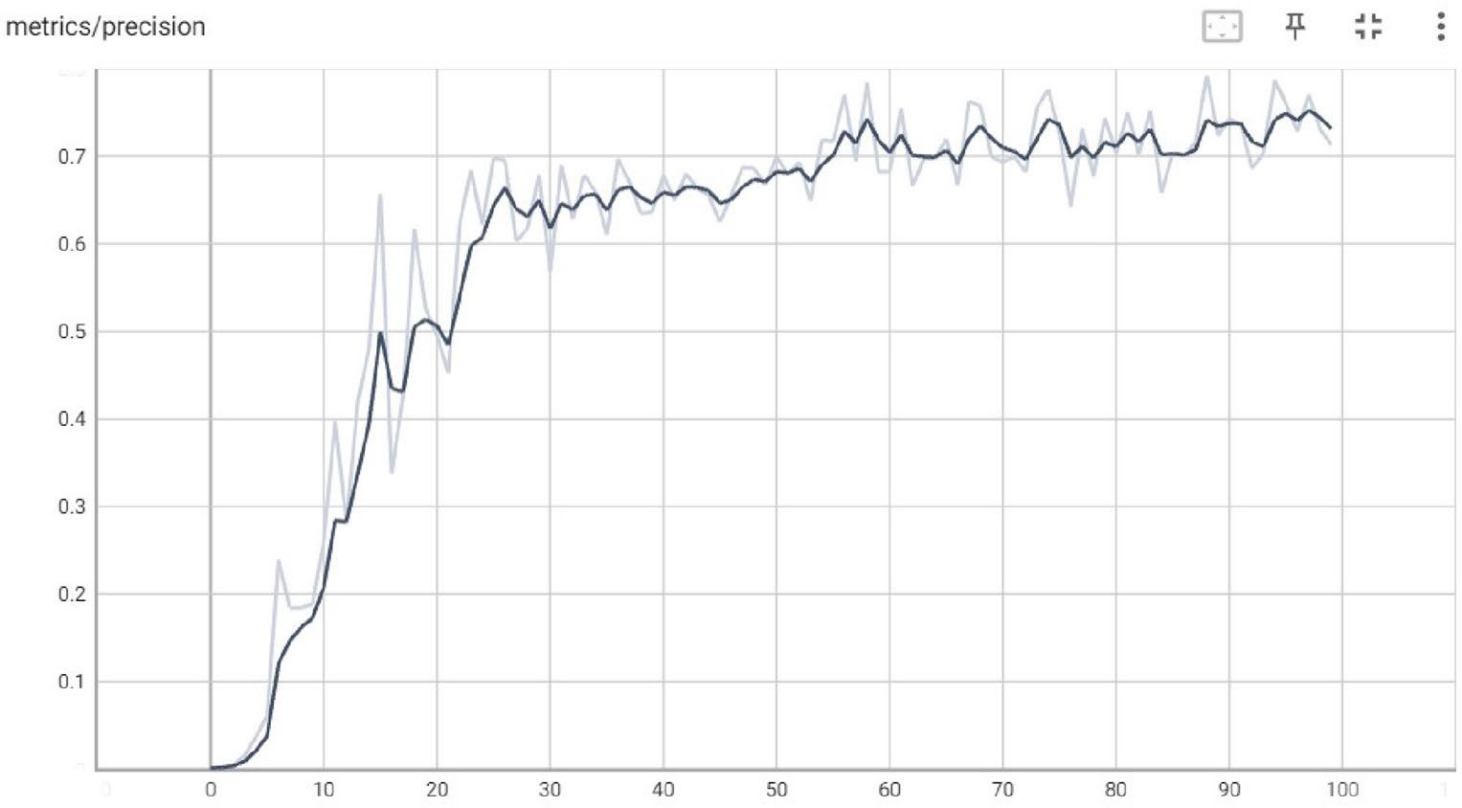
Precision gained by basic Yolov5 model.

The precision of a model is the degree to which its predictions are true. It is the proportion of optimistic predictions that turn out to be correct. Precision in the context of YOLO refers to the Model's success rate in properly identifying images of things. False positives are rare with a high-precision model since it only detects real items in the image. The x-axis represents the echo value, and the y-axis represents the precision value gained by the basic Yolov5 Model. Figure 6 demonstrates the precision values at different echos.

**Figure 7:**
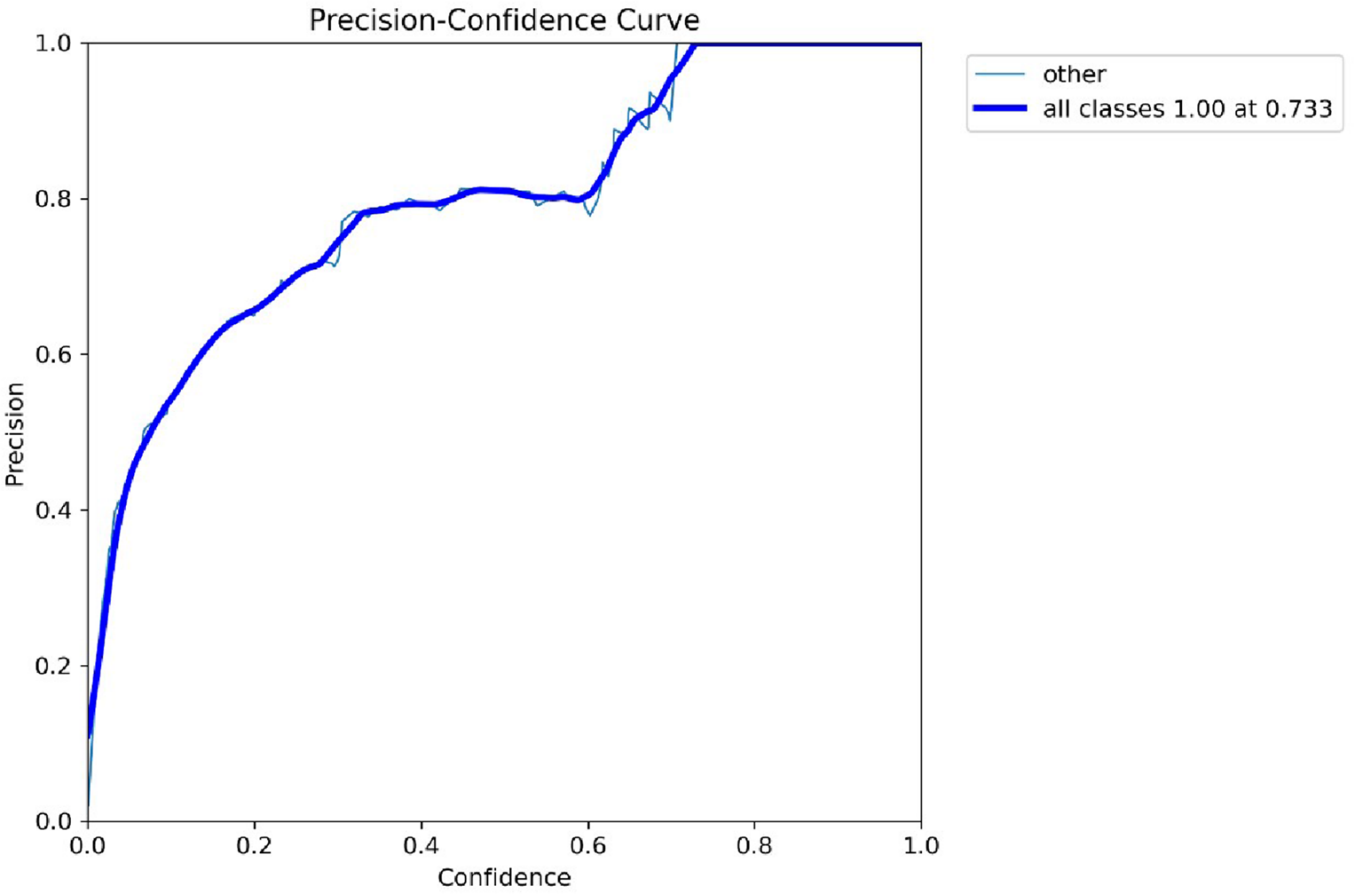
Precision-confidence curve by basic Yolov5 model.

For an object detection model, the values of precision and confidence can be graphically represented by a precision-confidence curve. The Model's projected bounding boxes are shown against their confidence ratings. In the context of a YOLO model, precision refers to the proportion of correct detections among all predictions. In contrast, confidence refers to the weight given by the Model to each predicted box's location inside the input image. Figure 7 demonstrates the relationship between Precision and Confidence values at different echos. The x-axis represents the Precision value, and the y-axis represents the Confidence value gained by the basic Yolov5 Model.

The Model's Accuracy at various levels of certainty can be graphically represented using a precision-confidence curve. All projected boxes are regarded as true positives at the curve's beginning, where the confidence threshold is set very low or at 1. The number of anticipated boxes and their Accuracy may fall with a rise in the confidence threshold. For every specific job, the curve may be used to determine the confidence level that best strikes a compromise between Accuracy and recall.

**Figure 8:**
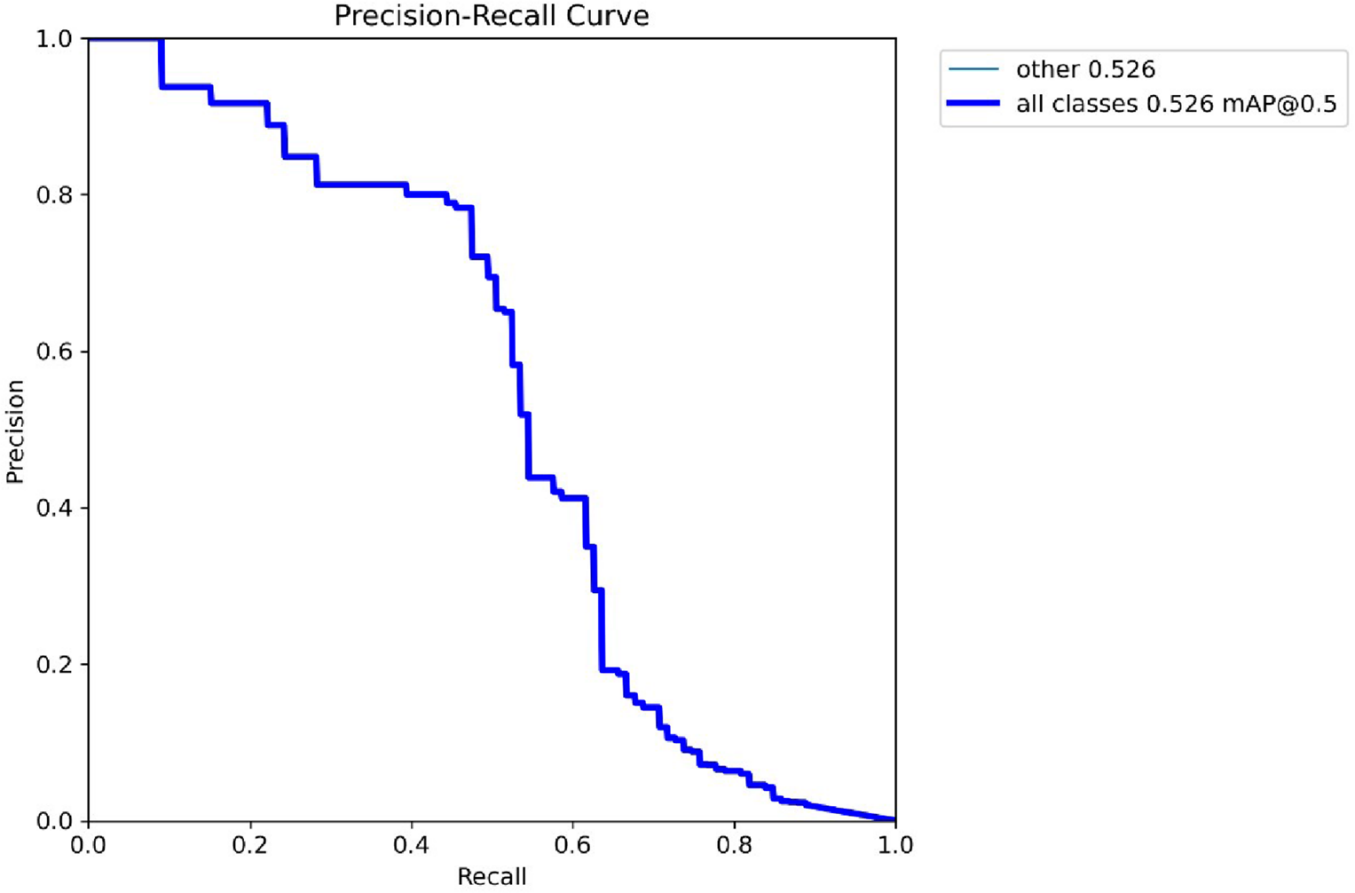
Precision-recall curve by basic Yolov5 model.

The strengths and shortcomings of a YOLO model may be better understood, as well as areas for improvement, by examining the Model's precision-confidence curve. Figure 8 demonstrates the relationship between Precision and Recall values at different echos. The x-axis represents the Recall value, and the y-axis represents the precision value gained by the basic Yolov5 Model.

**Figure 9:**
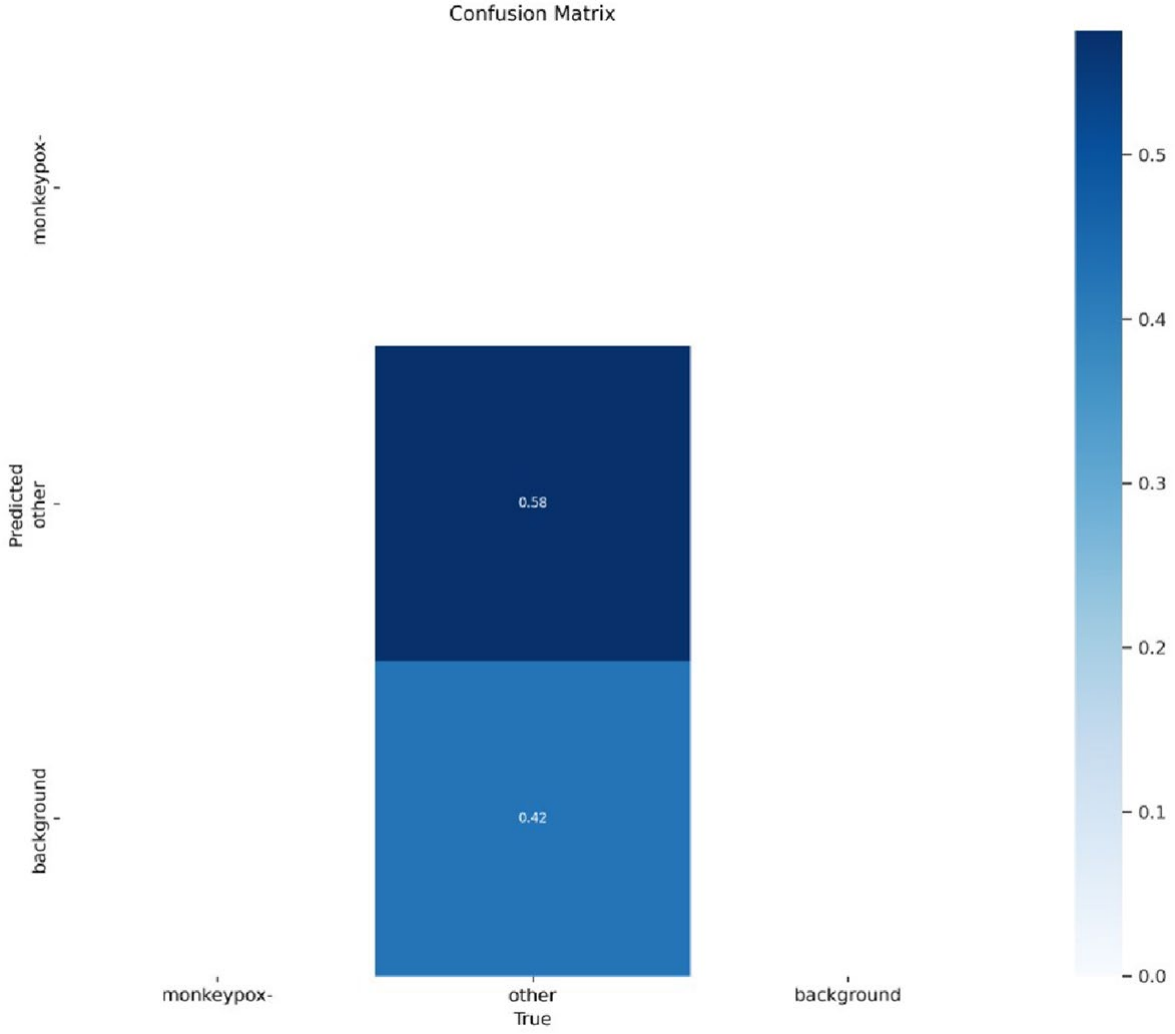
Confusion matrix.

Figure 9 depicts the confusion matrix in which the monkeypox's predicted and actual values are shown below.

### Experiment 2: modified Yolov5 model tuned with learning without forgetting (LwF)

**Figure 10:**
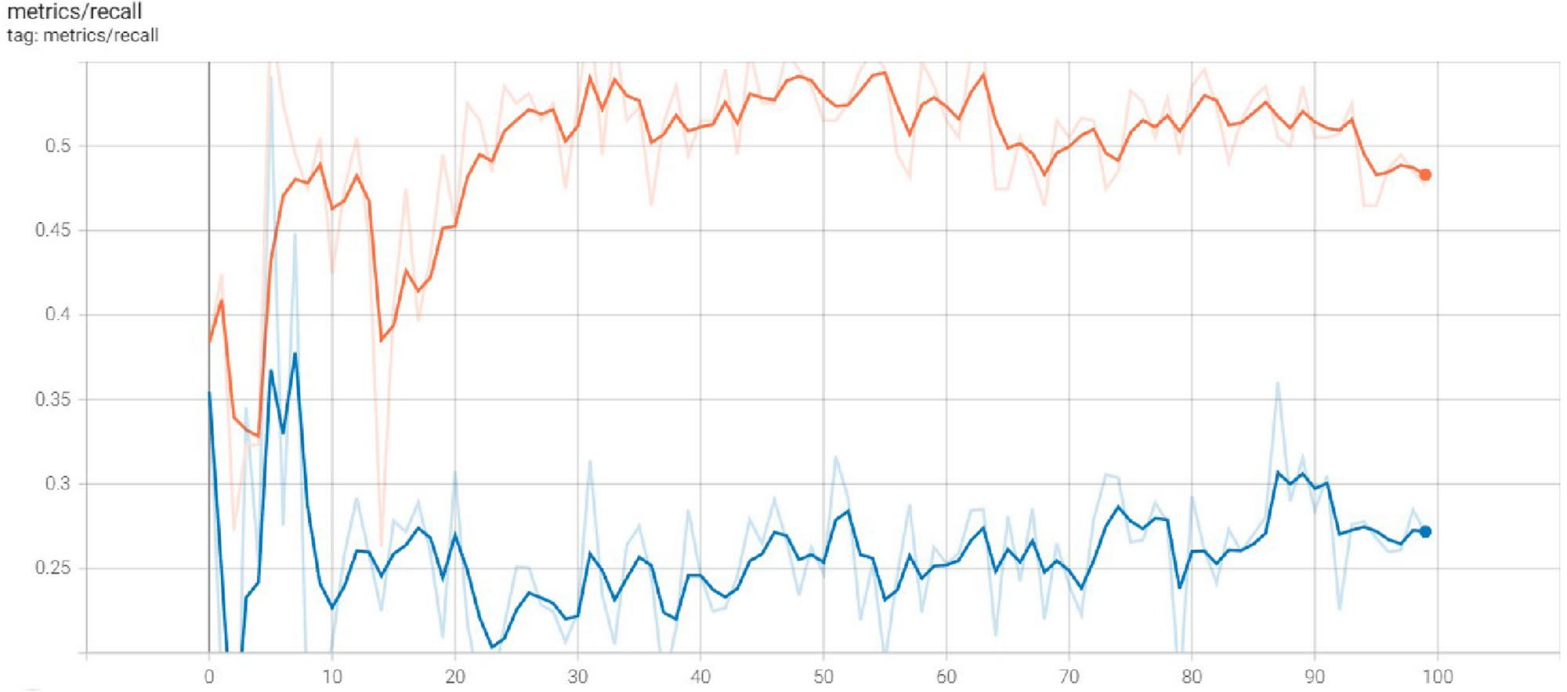
Recall gained by the basic Yolov5 model.

In Experiment 2, the authors executed the Modified Yolov5 Model tuned with Learning without Forgetting (LwF) on the prescribed Roboflow Monkeypox dataset on different 100 echos. Figure 10 demonstrates the recall values at distant echos. The x-axis represents the echo value, and the y-axis represents the recall value gained by the basic Yolov5 Model.

**Figure 11:**
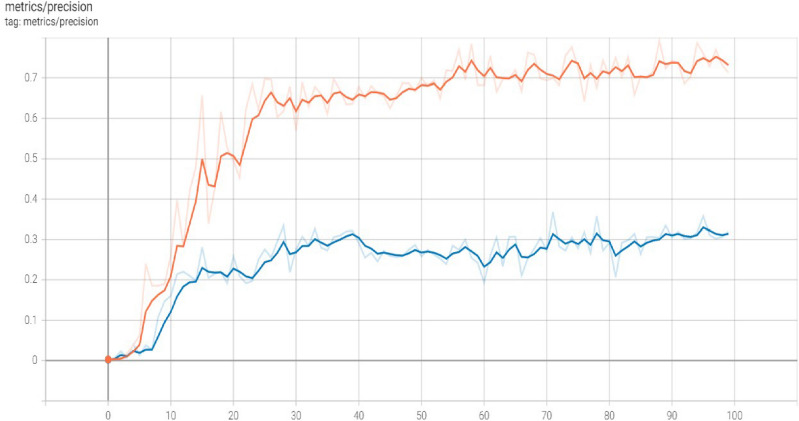
Precision gained by basic Yolov5 model.

Figure 11 demonstrates the precision values at different echos. The x-axis represents the echo value, and the y-axis represents the precision value gained by the basic Yolov5 Model.

**Figure 12:**
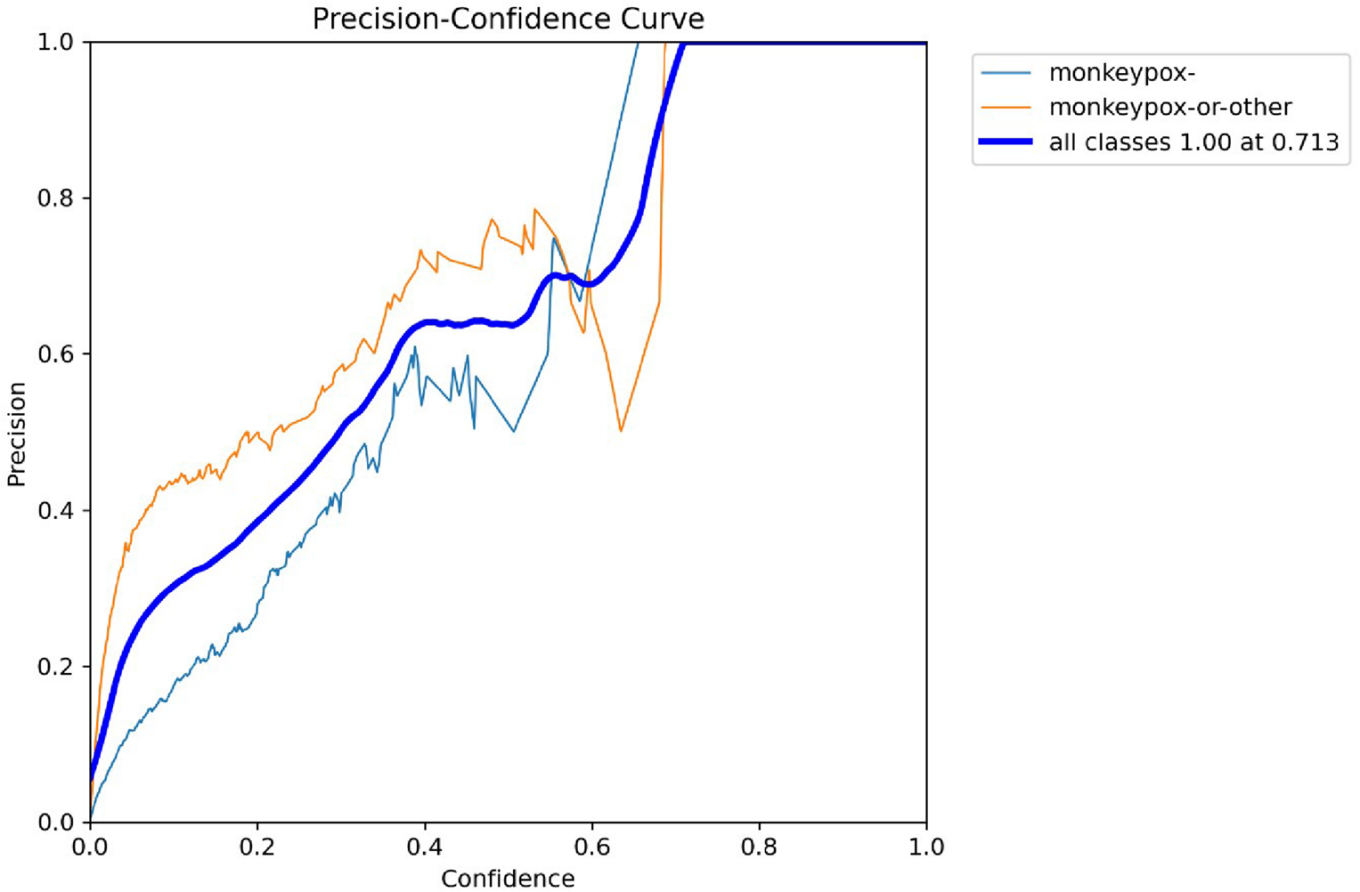
Precision-confidence curve by basic Yolov5 model.

Figure 12 demonstrates the relationship between Precision and Confidence values at different echos. The x-axis represents the Precision value, and the y-axis represents the Confidence value gained by the basic Yolov5 Model.

Table 2 demonstrates the classification measure for different optimizers (SGD, Bayesian and LwF optimizers) with the Yolo model in classifying the weather conditions.

**Table 2:** Classification measure.

Optimizer	Images	Precision	Recall	mAP@.5	mAP@.5:.95
SGD optimizer	971	0.756	0.684	0.456	0.199
Bayesian optimizer	971	0.676	0.569	0.495	0.354
Optuna optimizer	971	0.991	0.928	0.887	0.156

**Figure 13:**
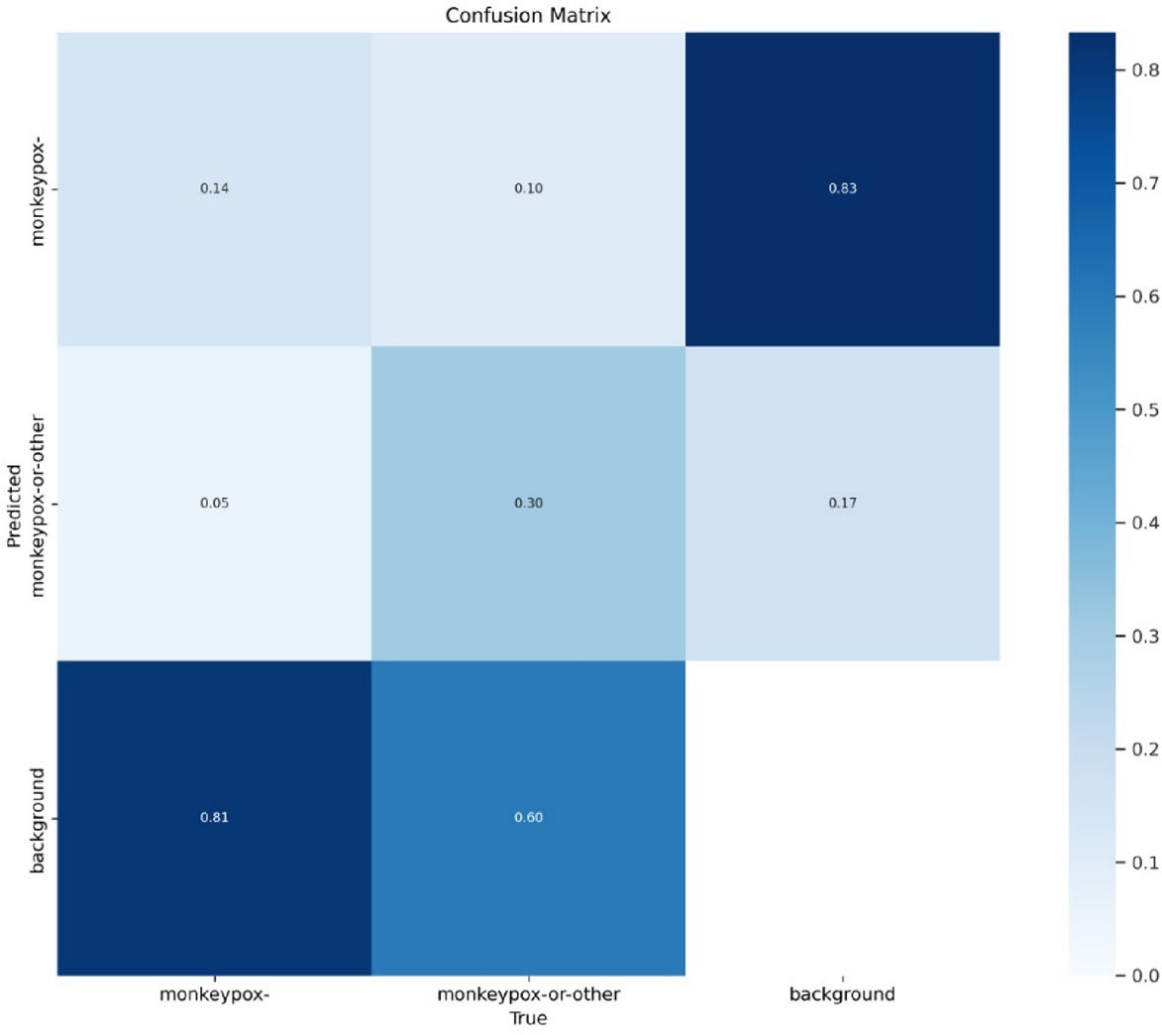
Confusion matrix.

Figure 13 shows the confusion matrix for the monkeypox disease classification. This figure depicts the actual and predicted values of background, monkeypox and monkeypox-or-other attributes.

**Figure 14:**
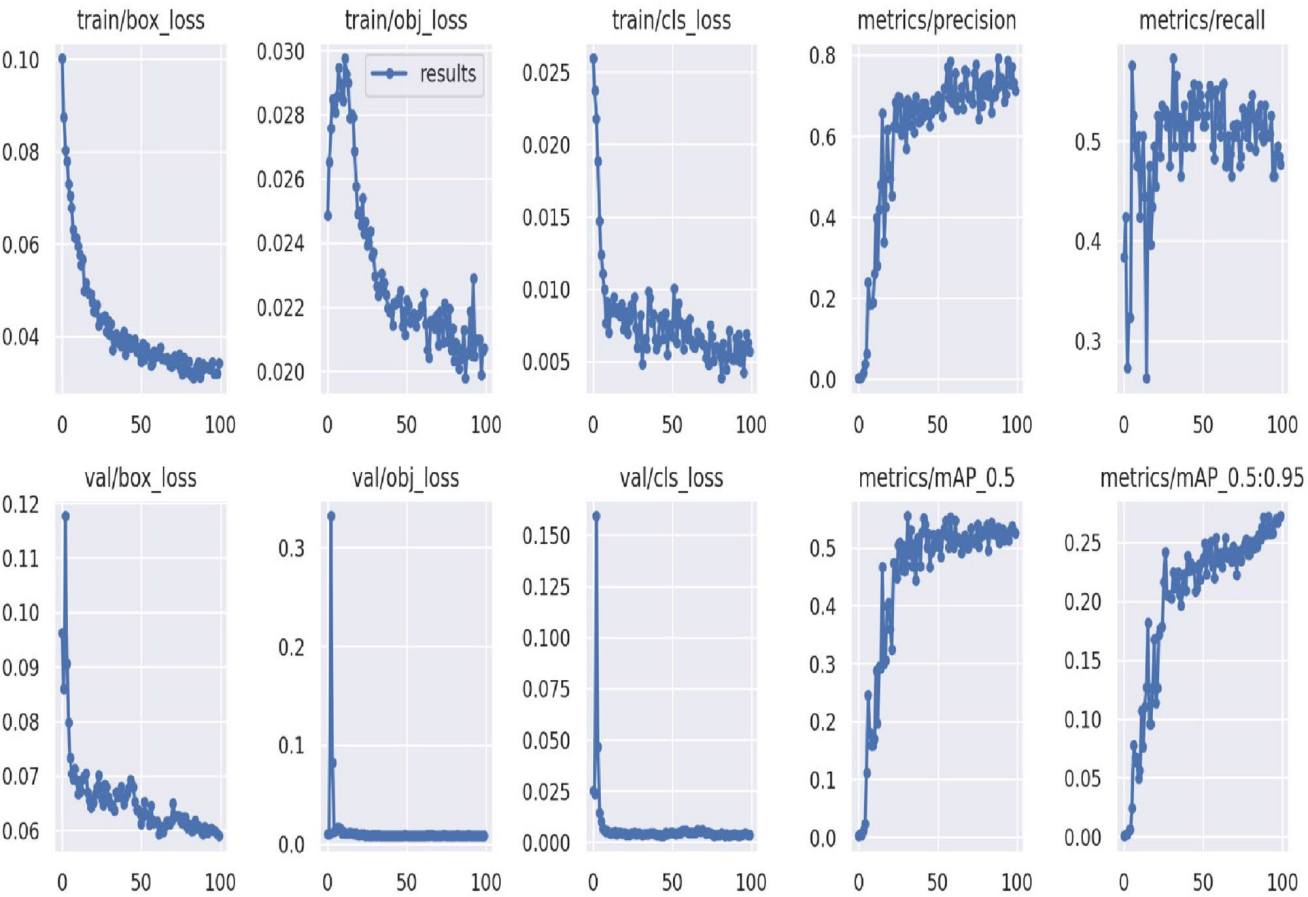
Performance of proposed model.

Finally, we show the proposed Model's overall performance in terms of box_loss, obj_loss, cls_loss, mAP_0.5, mAP_0.5:0.95, precision and recall in Fig. 14 below.

In Fig. 14, the bounding box predictions are coupled with a loss, denoted by box_loss. How effectively a model can locate and then categorize regions of interest in medical pictures, such as skin lesions or other signs of the disease, is a major component in deciding how successfully YOLOv5 can be used for human Monkeypox disease classification. The level of box_loss for proposed model is 0.06 after 100^th^ epoch. Objectness loss is critical for the efficient and accurate detection of areas in medical photos that may have indications of Monkeypox sickness during the classification process using YOLOv5. The diagnostic power of the model is improved by having a well-optimized objectness loss, making it a valuable resource for doctors. The level of obj_loss for proposed model is 0.020 after 100^th^ epoch. By fine-tuning the model's hyperparameters, we may be able to make more precise and fast diagnoses.

The loss in classification accuracy is often calculated as a cross-entropy measure between the anticipated class probabilities and the true class labels (one-hot encoded). How closely the probability distribution predicted by the model matches the real distribution is quantified by the loss. Lower classification accuracy, and hence less clinical value, would result from a large classification loss. Bad categorization show up as poor accuracy and recall scores, which might lead to a low F1-score and render the model unsuitable for clinical usage. Accuracy performance metrics for several approaches, including the suggested method, are shown in Table 3. The recommended Model was established to achieve more Accuracy than preexisting models.

**Table 3:** Result comparison.

S. No	Method	Performance
1	DenseNet201	93.1% Accuracy
2	ResNet5 model	87% Accuracy
3	GoogLeNet model	88.27% Accuracy
4	MobileNetv2 model	91.11% Accuracy
5	Pre-trained deep learning models	82.96(± 4.57%) Accuracy
6	SVM model	93.48% Accuracy
7	Weighted Naïve Bayes (WNB)	92.56% Accuracy
8	Yolov3 model	93.16% Accuracy
9	Yolo4 model	95.19% Accuracy
10	Proposed model	98.28% Accuracy

The result shows that the proposed Model gained 98.28% accuracy, which is more efficient than existing models.

## Discussion

Developing a deep learning model for the early detection of human monkeypox is a difficult but crucial challenge. Early illness detection using deep learning models is a promising area of research, and Human Monkeypox is no exception. Early detection of diseases is essential for successful treatment and management, and this is made possible through the use of deep learning models applied to medical picture analysis. Data gathering and preprocessing is the starting point for model construction. Gathering a collection of photos of people with and without Human Monkeypox Disease and preprocessing them such that they are all the same size and resolution is required. The Model's efficacy is heavily dependent on the quality and size of the Dataset; thus, this process is crucial. The next step is choosing an appropriate deep learning model for the classification job. For picture classification applications like identifying Human Monkeypox Disease, Convolutional Neural Networks (CNNs) are frequently employed. This problem is equally amenable to Residual Networks (ResNets) and Inception Networks.

After settling on a model, it's time to fine-tune the Model's hyper-parameters for maximum effectiveness. The hyper-parameters may be tuned using methods like Grid Search, Random Search, and Bayesian Optimization. The learning rate, batch size, number of layers, filters, and dropout rate are all hyper-parameters that may be adjusted. Careful adjustment of these hyper-parameters is required to prevent either overfitting or underfitting. After the hyper-parameters have been modified, the Model may be trained on the preprocessed Dataset. Methods like early halting and data augmentation can be used to reduce the likelihood of overfitting. The Model's strength and generalizability may also be tested through cross-validation and confusion matrix analysis. Data collection and preprocessing, model selection, hyperparameter tuning, training the Model, evaluating its performance, and finally deploying it into production are all crucial steps in developing an optimized hyperparameter-tuned deep learning model for Human Monkeypox Disease Detection. We can construct a robust and precise model for the early identification of Human Monkeypox Disease if we follow these steps properly. Human monkeypox disease detection using deep learning models with improved hyper-parameter tuning is an area with much room for future investigation and advancement. Prospective future focuses are listed below. Most human monkeypox disease detection algorithms are currently trained with only medical photos^32^.

Using previously trained models as a foundation for developing new models is the essence of transfer learning, a potent learning approach. More precise and time-saving models for identifying human monkeypox cases can be developed in the future with the help of transfer learning. Explainable AI methods can enhance the interpretability of deep learning models. This is especially significant in medical applications, where it is crucial to comprehend the reasoning behind the Model's diagnosis. The detection of human monkeypox disease can be further explored in the context of building explainable AI approaches. To identify Human Monkeypox in real-time, deep learning models must be used to do real-time picture analysis. This can be especially helpful in outlying places with limited medical care options. With the proliferation of new data sources, deeper learning models may be trained on ever-growing pools of information. This can enhance the Model's Accuracy and resilience, making it better equipped to diagnose Human Monkeypox Disease. There is hope for the application of hyper-parameter tailored deep learning models in the detection of Human Monkeypox. Improved public health and safety models may be developed using cutting-edge methods such as multi-modal data, transfer learning, explainable AI, real-time detection, and bigger datasets.

The following are some limitations of a hyperparameter-tuned deep learning model for accurate human Monkeypox illness detection:

- Large datasets are typically necessary for efficient training of deep learning models. When a model is trained with a small sample of data, it may not accurately represent the larger population.
- Sometimes there are more samples of healthy people than those with Monkeypox in medical databases. This might cause bias in the training process for the model.
- It is common for hyperparameter tweaking to consume a large amount of processing time. This might be a problem for organizations that lack easy access to such materials.
- The model's computational efficiency and scalability to bigger datasets are unknown, despite the possibility that it performs well on the current dataset.
- While hyperparameter adjustment can reduce the likelihood of overfitting, it is still possible, especially in complicated models.
- As “black boxes”, deep learning models are sometimes criticized for making it impossible to comprehend their decision-making process, which is especially important in medical applications.

## Conclusions

In this study, we aimed to foresee the spread of monkeypox and provide a global summary of the disease's prevalence to raise awareness. Moreover, we spoke about some of the most critical factors contributing to it. Using the presented Model, we looked into making more precise predictions about the spread of monkeypox. There was a lot of volatility in the little Dataset used for the monkeypox investigation. This study had several problems, such as the inherent limitations of extending results from a single study, the difficulty in obtaining appropriate measurements, and the struggle to select a suitable model to represent the data^33^. The results would be more reliable if the Dataset were more extensive and the variance was more minor^34,35^.

In healthcare, public health management, and even economic security, society as a whole stands to profit greatly from the use of a hyperparameter-tuned deep learning model for the identification of human Monkeypox. Some advantages of this method are listed below.

### Early detection and treatment

- Predictive Analysis: The key to successful therapy is early diagnosis. Medical intervention can be made sooner if diagnostics are improved by model tuning.
- Reduced Mortality Rate: If the disease is diagnosed and treated quickly, the fatality rate from monkeypox may be lowered.

### Resource allocation

- Utilization of Healthcare Resources: By eliminating the need for human intervention in the detection process, healthcare providers would be able to use their time and energy more productively toward patient care.
- Cost Savings in Health Care: With the help of automation and improved diagnostic accuracy, healthcare may be provided at a lower cost to more people.

### Epidemiological benefits

- Outbreak Control: To prevent the further spread of an infectious illness, rapid and precise detection is essential.
- Decision Making Based on Data: Data generated by trustworthy detection algorithms can aid epidemiologists and policymakers in making informed decisions about public health.

### Technology advancements

- Stimulates Further Research: The creation and validation of such a model has the potential to fuel more investigation into the use of AI in healthcare, ultimately leading to more significant technical advances.
- Scalability: Once these models have been created and proven effective for one illness or area of healthcare, they may be expanded to help a much larger population.

### Ethical and social impact

- Equity in Health Care: By deploying a calibrated, accurate model in these hard-to-reach places, we can help more people get the treatment they need.
- Public Awareness: An uptick in illness awareness generally follows the introduction of such tools, and this in turn motivates people to take better care of their health.

### Effect on the economy

- Financial Calmness: An outbreak’s negative social and economic effects, such as a loss of labor, decreased productivity, and increased healthcare expenditures, can be lessened by early diagnosis and efficient control.

### Global health security

- Worldwide Preparation: The early diagnosis and control of infectious illnesses with international transmission potential, such as monkeypox, is an important part of maintaining international health security.
- International Collaboration: Sharing or adapting the technologies on a global scale promotes coordinated worldwide action against illness.

Whether for individual patient care or global public health management, the proposed hyperparameter-tuned deep learning technique for successful human Monkeypox illness diagnosis might represent a major step forward in healthcare technology.

The Human Monkeypox Virus may be detected using the well-known deep-learning model YOLOv5. However, picking the best hyperparameters for YOLOv5 might be difficult, so Optuna is a helpful tool. The optimal YOLOv5 model hyperparameters may be quickly found using Optuna's TPE method and a well-defined objective function. Another interesting strategy for enhancing the precision of Human Monkeypox Disease diagnosis is using a multilayer ResNet architecture with Adadelta optimization. ResNet-18, ResNet-34, and ResNet-50 may be combined to use the best features of each Model and outperform the performance of a single ResNet design. The Accuracy of Human Monkeypox Disease identification may be greatly improved by combining YOLOv5 with Optuna for hyperparameter tweaking and the multiple ResNet architecture with Adadelta optimization.

Medical imaging can benefit greatly from object identification utilizing deep learning models like YOLO for illness diagnosis. YOLOv5 can potentially enhance the speed and precision of monkeypox diagnoses. The YOLOv5 model’s performance on the monkeypox dataset can be improved by hyperparameter tweaking with Optuna. Optuna employs the TPE algorithm, which effectively searches for the optimal hyperparameters to maximize a specified objective function. However, the quality and amount of the Dataset utilized for training can significantly impact the YOLO model's performance. The Model's clinical utility may also be diminished if it cannot successfully apply to data that does not match those of the training and validation sets.

Hyperparameter tweaking using Optuna and using YOLO for detecting monkeypox show promise for enhancing diagnostic Accuracy and efficiency, but further study is needed before they can be used in a clinical context.

## Data Availability

The Dataset has been the Roboflow Dataset Repository. The link to the prescribed Dataset is https://universe.roboflow.com/monkeypox-o7ktt/monkeypox-detection-lym6c. This Dataset consists of 971 images, with 849 pictures used as the Training Set, 81 as the Validation Set, and the remaining photos used in the Testing Set.
